# The integrative oncology supplement—a paradigm for both patient care and communication

**DOI:** 10.3747/co.v15i4.267

**Published:** 2008-08

**Authors:** S.M. Sagar

## FOREWORD

Integrative oncology is both a science and a philosophy that focuses on the complexity of the health of cancer patients and proposes a multitude of approaches to accompany the conventional therapies of surgery, chemotherapy, molecular therapeutics, and radiotherapy to facilitate health.

—*Stephen Sagar*

The editorial that follows departs from our usual expository style in favour of an introduction from Dr. Stephen Sagar of a very special supplement to *Current Oncology.* We trust that you will enjoy reading the upcoming supplement on integrative oncology, whose plethora of contributions from talented practitioners is being published in a combination of hard copy and online articles at the *Current Oncology* Web site, www.current-oncology.com. Furthermore, I hope you can find the time to respond to Dr. Sagar’s appeal in the supplement to add your voice to the unique follow-up discussion that will be hosted at the Web site.

M. McLean md

The concept “integrative” is defined as “to join with something else,” “unite,” “make part of a larger unit,” and be “open to people with various cultural values as equals” 1. One of the fundamental tenets of integrative oncology 2,3 is to consider the cultural values of patients and to incorporate those values into the decision plan, using various self-empowerment tools that are safe, that improve outcome and that are, preferably, cost-effective. Conceptually, the same is true for education and learning. The term “integrative learning” was coined by Jerry Perez de Tagle 4 and comes in many varieties: connecting skills and knowledge from multiple sources and experiences, applying skills and practices in various settings, making use of diverse and even contradictory points of view, and understanding issues and positions contextually.

Thanks to a generous grant from the Lotte and John Hecht Memorial Foundation 5, a special supplement of *Current Oncology* on integrative oncology is being printed concurrently with the regular issue. The Foundation continues the principles of its founders to integrate diverse societal values and has taken a particular interest in the investigation and evaluation of complementary and alternative medicine (cam), particularly as part of a cancer treatment program. In line with the principles of integration, the *Current Oncology* supplement will incorporate peer-reviewed manuscripts that focus on cam, plus educational articles on integrative oncology services and research. This material will be presented in a hybrid “integrative” format that uses the resources both of print and of the Internet, including appropriate links, colour photographs, video streams, and lectures accompanied by slides.

In his publication “The New Alchemy: Transmuting Information into Knowledge in an Electronic Age,” Professor Alejandro Jadad eloquently discusses the transition into the new age of media communications and the integration of the new media into health care 6. In that article, he concludes, “[T]his is meant to be an interactive feature.... [T]he new media and tools to which we are being exposed will undoubtedly change the way in which we communicate, learn and think.” Those media are currently the ones in which our patients are exploring information—in a virtual world with no boundaries. With structure, guidance, and the separation of fact from fiction, the Internet is a powerful tool for education and decision-making. However, communication and integration of virtual information requires guidance based on knowledge derived from evidence. In the integrative oncology supplement of *Current Oncology,* my co-editor (Anne Leis of the University of Saskatchewan) and I have integrated these concepts, especially as they relate to cancer patients utilizing cam.

Part of the impetus for the supplement was the conference titled Integrating Wellness into Cancer Care, held at the University of Toronto, October 4–5, 2007. The conference was led by Dr. Paul Fortin, in memory of his wife, Dr. Veronique Benk. Veronique was a radiation oncologist, clinician, and researcher who specialized in breast cancer, and she was devoted to her patients. Her personal experience of breast cancer and myeloid leukemia was transformative, and she embraced a wider approach to cancer treatment. Her approach integrated state-of-the-art medical care with a new emphasis on spirituality, wellness, and quality of life.

From that point, a host of distinguished authors contributed their knowledge and research to the supplement—Lynda Balneaves, Alison Brazier, Alastair Cunningham, Gary Deng, Meghan Duncan, Jane Maher, Doreen Oneschuk, Dugald Seeley, Simon Sutcliffe, Mary Vachon, Marja Verhoef, Raimond Wong, and Anne Leis and I. We hope that you will find the issue stimulating and thought-provoking.
